# Incorporating radiomics into clinical trials: expert consensus endorsed by the European Society of Radiology on considerations for data-driven compared to biologically driven quantitative biomarkers

**DOI:** 10.1007/s00330-020-07598-8

**Published:** 2021-01-25

**Authors:** Laure Fournier, Lena Costaridou, Luc Bidaut, Nicolas Michoux, Frederic E. Lecouvet, Lioe-Fee de Geus-Oei, Ronald Boellaard, Daniela E. Oprea-Lager, Nancy A Obuchowski, Anna Caroli, Wolfgang G. Kunz, Edwin H. Oei, James P. B. O’Connor, Marius E. Mayerhoefer, Manuela Franca, Angel Alberich-Bayarri, Christophe M. Deroose, Christian Loewe, Rashindra Manniesing, Caroline Caramella, Egesta Lopci, Nathalie Lassau, Anders Persson, Rik Achten, Karen Rosendahl, Olivier Clement, Elmar Kotter, Xavier Golay, Marion Smits, Marc Dewey, Daniel C. Sullivan, Aad van der Lugt, Nandita M. deSouza, European Society of Radiology

**Affiliations:** 1 PARCC, INSERM, Radiology Department, AP-HP, Hopital europeen Georges Pompidou, Université de Paris, F-75015 Paris, France; 2 European Imaging Biomarkers Alliance (EIBALL), European Society of Radiology, Vienna, Austria; 3 Imaging Group, European Organisation of Research and Treatment in Cancer (EORTC), Brussels, Belgium; 4 School of Medicine, University of Patras, University Campus, Rio, 26 500 Patras, Greece; 5 College of Science, University of Lincoln, Lincoln, LN6 7TS UK; 6 Department of Radiology, Institut de Recherche Expérimentale et Clinique (IREC), Cliniques Universitaires Saint Luc, Université Catholique de Louvain (UCLouvain), B-1200 Brussels, Belgium; 7 Department of Radiology, Leiden University Medical Center, Leiden, The Netherlands; 8 Biomedical Photonic Imaging Group, University of Twente, Enschede, The Netherlands; 9 Department of Radiology & Nuclear Medicine, Cancer Centre Amsterdam, Amsterdam University Medical Centers (VU University), Amsterdam, The Netherlands; 10 Quantitative Imaging Biomarkers Alliance, Radiological Society of North America, Oak Brook, IL USA; 11 Department of Quantitative Health Sciences, Cleveland Clinic, Cleveland, OH USA; 12 Department of Biomedical Engineering, Istituto di Ricerche Farmacologiche Mario Negri IRCCS, Bergamo, Italy; 13 Department of Radiology, University Hospital, LMU Munich, Munich, Germany; 14 Department of Radiology & Nuclear Medicine, Erasmus MC, University Medical Center, Rotterdam, The Netherlands; 15 Division of Cancer Sciences, University of Manchester, Manchester, UK; 16 Department of Biomedical Imaging and Image-guided Therapy, Medical University of Vienna, Vienna, Austria; 17 Department of Radiology, Centro Hospitalar Universitário do Porto, Instituto de Ciências Biomédicas de Abel Salazar, University of Porto, Porto, Portugal; 18 Quantitative Imaging Biomarkers in Medicine (QUIBIM), Valencia, Spain; 19 Nuclear Medicine, University Hospitals Leuven, Leuven, Belgium; 20 Nuclear Medicine and Molecular Imaging, Department of Imaging and Pathology, KU Leuven, Leuven, Belgium; 21 Division of Cardiovascular and Interventional Radiology, Dept. for Bioimaging and Image-Guided Therapy, Medical University of Vienna, Vienna, Austria; 22 Department of Radiology and Nuclear Medicine, Radboud University Medical Center, 6525 GA Nijmegen, The Netherlands; 23 Radiology Department, Hôpital Marie Lannelongue, Institut d’Oncologie Thoracique, Université Paris-Saclay, Le Plessis-Robinson, France; 24 Nuclear Medicine, Humanitas Clinical and Research Hospital – IRCCS, Rozzano, MI Italy; 25 Imaging Department, Gustave Roussy Cancer Campus Grand, Paris, UMR 1281, INSERM, CNRS, CEA, Universite Paris-Saclay, Saint-Aubin, France; 26 Department of Radiology, and Department of Health, Medicine and Caring Sciences, Center for Medical Image Science and Visualization (CMIV), Linköping University, Linköping, Sweden; 27 Department of Radiology and Medical Imaging, Ghent University Hospital, Gent, Belgium; 28 Department of Radiology, University Hospital of North Norway, Tromsø, Norway; 29 Department of Radiology, University Medical Center Freiburg, Freiburg, Germany; 30 Queen Square Institute of Neurology, University College London, London, UK; 31 Department of Radiology, Charité Universitätsmedizin Berlin, Berlin, Germany; 32 Dept. of Radiology, Duke University, 311 Research Dr, Durham, NC 27710 USA; 33 Division of Radiotherapy and Imaging, The Institute of Cancer Research and Royal Marsden NHS Foundation Trust, London, UK; 34 Am Gestade 1, 1010 Vienna, Austria

**Keywords:** Radiology, Statistics and numerical data, Standardization, Validation studies, Clinical trial

## Abstract

**Abstract:**

Existing quantitative imaging biomarkers (QIBs) are associated with known biological tissue characteristics and follow a well-understood path of technical, biological and clinical validation before incorporation into clinical trials. In radiomics, novel data-driven processes extract numerous visually imperceptible statistical features from the imaging data with no a priori assumptions on their correlation with biological processes. The selection of relevant features (radiomic signature) and incorporation into clinical trials therefore requires additional considerations to ensure meaningful imaging endpoints. Also, the number of radiomic features tested means that power calculations would result in sample sizes impossible to achieve within clinical trials. This article examines how the process of standardising and validating data-driven imaging biomarkers differs from those based on biological associations. Radiomic signatures are best developed initially on datasets that represent diversity of acquisition protocols as well as diversity of disease and of normal findings, rather than within clinical trials with standardised and optimised protocols as this would risk the selection of radiomic features being linked to the imaging process rather than the pathology. Normalisation through discretisation and feature harmonisation are essential pre-processing steps. Biological correlation may be performed after the technical and clinical validity of a radiomic signature is established, but is not mandatory. Feature selection may be part of discovery within a radiomics-specific trial or represent exploratory endpoints within an established trial; a previously validated radiomic signature may even be used as a primary/secondary endpoint, particularly if associations are demonstrated with specific biological processes and pathways being targeted within clinical trials.

**Key Points:**

*• Data-driven processes like radiomics risk false discoveries due to high-dimensionality of the dataset compared to sample size, making adequate diversity of the data, cross-validation and external validation essential to mitigate the risks of spurious associations and overfitting.*

*• Use of radiomic signatures within clinical trials requires multistep standardisation of image acquisition, image analysis and data mining processes.*

*• Biological correlation may be established after clinical validation but is not mandatory.*

## Introduction

Quantitative imaging biomarkers (QIBs) are associated with tissue characteristics that are altered by disease and its treatment. Necrosis decreases tissue cellularity and increases water content manifesting as an increase in T2 [1], a reduction in glucose uptake [2] and an increase in elasticity [3]. Perfusion imaging detects and characterises hypervascular lesions such as cancers, or monitors the effect of anti-angiogenic drugs [4, 5]. Implementation of QIBs into clinical trials follows a well-defined path from discovery, through a process of technical and biological validation, to implementation and clinical validation. A roadmap defining the process was published as a consensus statement from multiple stakeholders [6]. Despite this, QIBs have been slow to be adopted as trial endpoints because of the relative complexity of imaging protocols and variability of the quantified output under differing conditions (e.g. hardware, software, protocol and observer variability) [7].

**Fig. 1:**
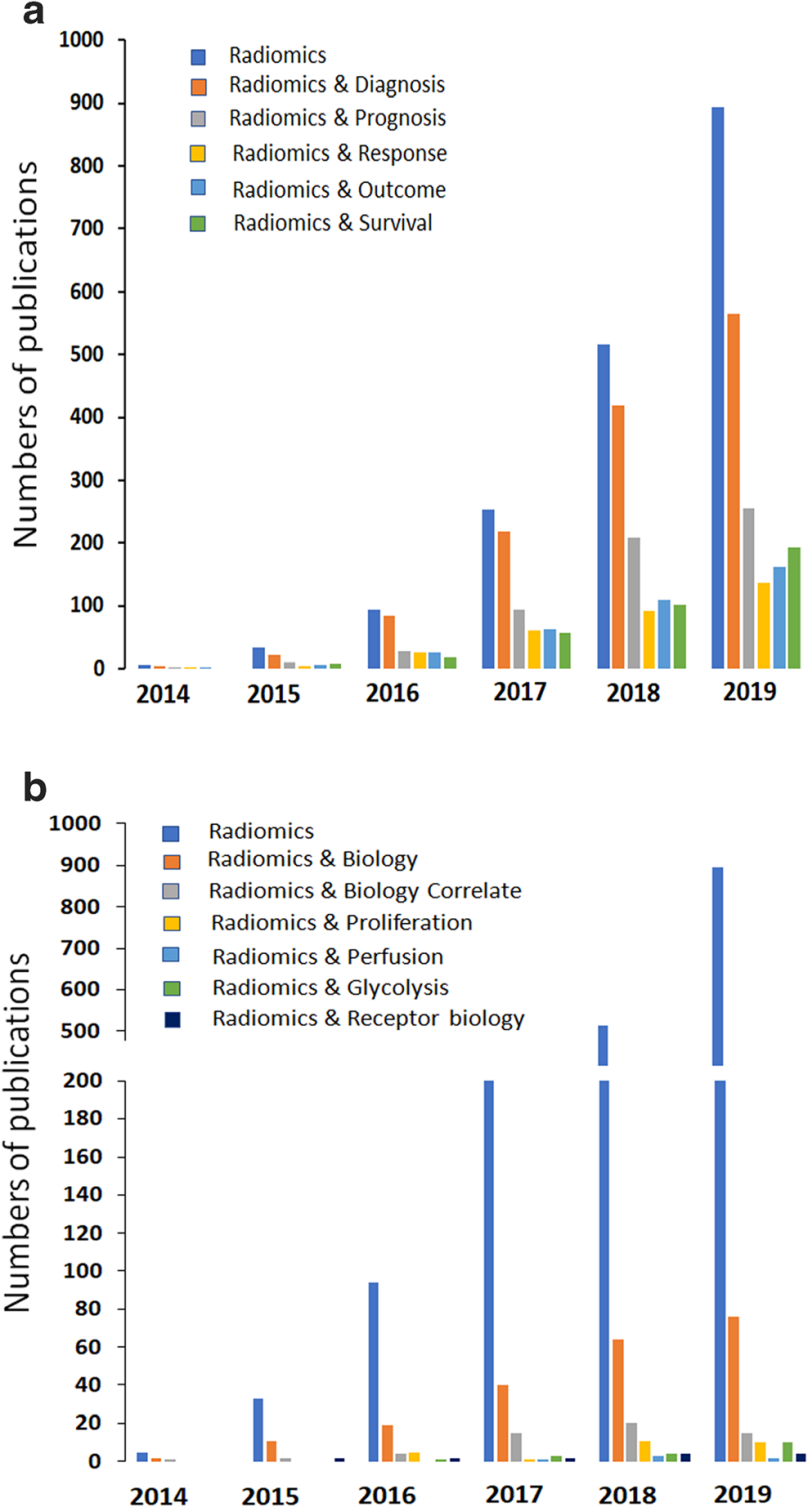
Increase in radiomics related publications over last 6 years (**a**) by patient status/outcome and (**b**) by biological association using data extracted from PubMed using the indicated MeSH terms. The exponential increase in radiomics publications relates mainly to usage as indicated in **a**, and not to their underlying biological associations as indicated in **b**

Recently, a new approach to derive imaging biomarkers has been advocated through the concept of radiomics [8, 9]. This data-driven framework ‘discovers’ quantitative information within images by extracting high-dimensional data (‘features’) beyond that visually perceptible, using computational statistics (often based on machine learning algorithms) to predict or establish association with a meaningful clinical endpoint [10, 11]. Technical and clinical performance of the ‘radiomic signature’ (specific combination of mathematically derived features) determines its appropriateness. If considered necessary, a link to a biological process is explored a posteriori [12]. Radiomic signatures have been associated with outcome or response [13], and may be used together with clinical, histological and genomic metrics as part of a nomogram of features [14]. The exponential rise in publications involving data-driven biomarkers has not been accompanied by a mechanism-based understanding of their nature but focuses on their ability to classify disease and patient outcome (Fig. 1). Radiomics has been used for detecting cancer [15], cancer staging [16], performing classifications [17], assessing response to chemotherapy [18], radiation therapies [19–22], immunotherapy [23–26] and predicting/prognosing survival [27].

A major disadvantage of a non-mechanistic data-driven approach is that random chance associations may occur. Most studies look at the associations between a large number of features extracted from discretised images and prognosis/response/outcome in an inadequate number of samples. For biomarker profiles that rely on statistical rather than biological associations, generalisation and scalability to multicentre trials requires more than a simple standardisation process. Also, their validation pathway needs to incorporate measures that may differ substantially from traditionally accepted methods. This article prepared by imaging experts from the European Society of Radiology EIBALL (European Imaging Biomarker ALLiance) and the EORTC (European Organisation for Research and Treatment of Cancer) Imaging Group with representatives from QIBA (Quantitative Imaging Biomarkers Alliance) examines how the process of standardising and validating data-driven imaging biomarkers differs from those based on biological associations, and what measures need to be considered when implementing them into clinical trials and, eventually, into clinical routine. Structured discussions were conducted via teleconferencing and written communications.

## Standardising the radiomics process for clinical trials

**Fig. 2:**
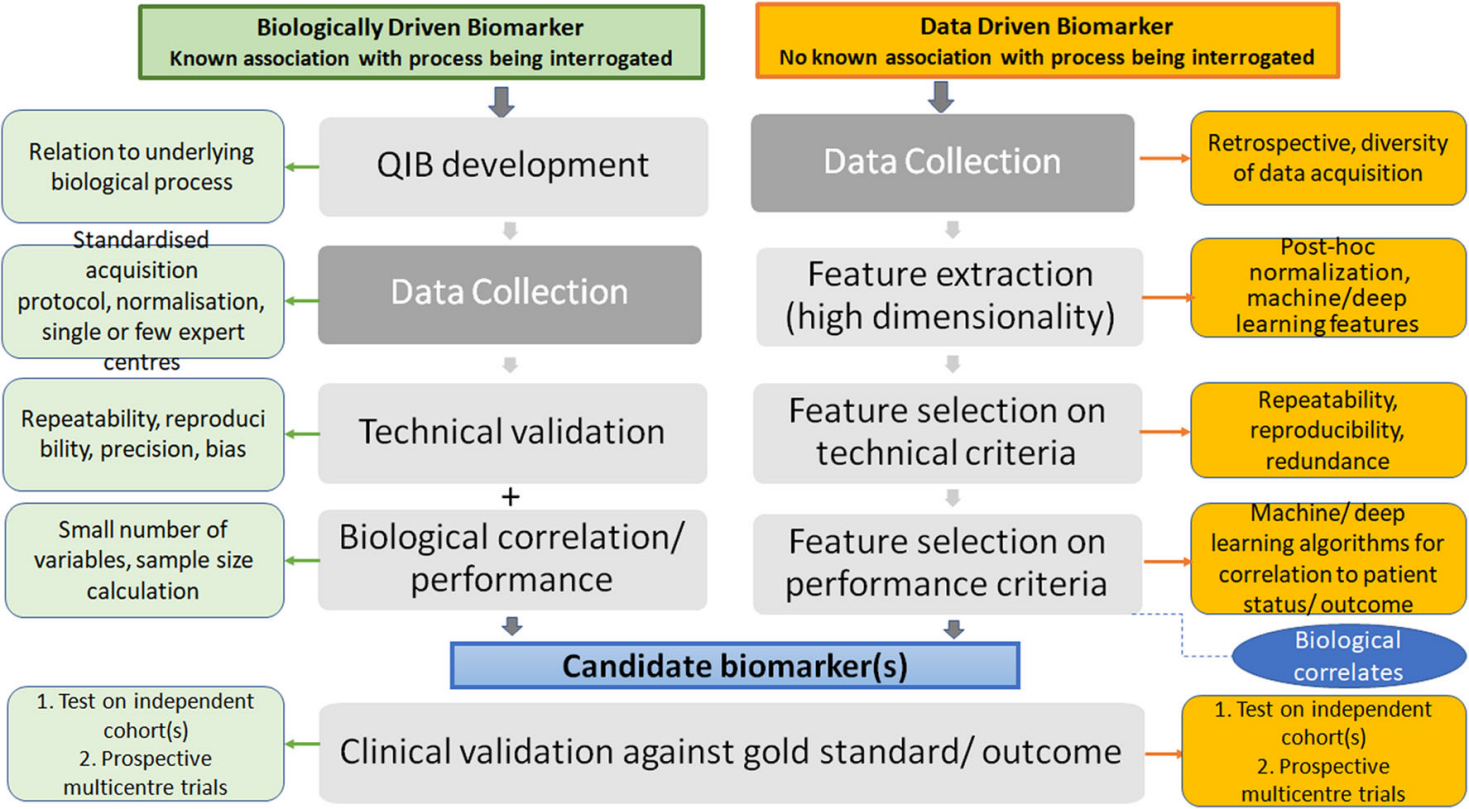
Pathways comparing processes required for biologically driven and data-driven biomarkers. Biologically driven biomarkers derived from known associations with a specific biological process require a specific predetermined acquisition protocol and image processing technique and involve technical, biological and clinical validation steps with recognised requirements (green boxes). Data-driven biomarkers assume that the statistical features that relate to the biological process or outcome are unknown so that all possible features are extracted from the images and steps to determine their technical and clinical performance are needed (orange boxes). Feature extraction and selection depend on the data mining process (machine and deep learning algorithms). A training dataset and validation dataset allow selection of most promising feature(s), and an independent test dataset allows evaluation of performance of imaging biomarker. Biological links are explored a posteriori

Radiomics analyses rely on image acquisition, image analysis and computational statistics [28], so standardisation of these domains is mandatory prior to their validation (Table 1). As radiomics analyses have been applied to CT [29–31], MRI [32–36], nuclear medicine using FDG-PET [37–42] and other tracers [43, 44], and ultrasound [45], image acquisition standardisation needs to consider modality, scanner and scan protocol. Standardisation of image analysis needs to consider software (consistency of technical implementation) and subjectivity (human interaction). Standardisation of computational statistics needs to consider adequacy, performance and requirements for validation of algorithms and models (Fig. 2).

**Table 1:** Comparison of standardisation steps for biologically driven and data-driven biomarkers (*QA*, quality assurance; *QC*, quality Control; *VOI*, volume of interest)

Steps	Biologically driven quantitative biomarkers	Data-driven quantitative biomarkers
Image acquisition	• Standardised protocols (single and multicentre) • QA/QC process across instruments, sites • Stability of measurement monitored with phantom studies; may be strengthened by human subject test-retest	• Non-standardised protocols in discovery phase followed by standardised protocols within trials • QA/QC process across instruments, sites • Stability of measurement requires human subject test-retest
VOI delineation	• Can be manual or semi-automated • Can be machine-learnt • Deep learning available but infrequently used	• Can be manual or semi-automated • Can be machine-learnt • Can be derived from fully convolutional neural networks
Data analysis	• Commercial or academic software applicable to datasets regardless of their source	• Algorithms used are specific to image datasets and may require adaptation and standardisation for individual situations or new datasets*
Biomarker extraction	• Follows standard formula that describes the biological feature (e.g. tissue density, perfusion, diffusion, standardised uptake of radiotracers related to a biological process/receptor status)	• Algorithm-based mathematical feature extraction not directly linked to a biological process, followed by selection of feature combination that best separate disease from no disease, good from poor outcome (e.g. shape features such as diameter, sphericity; histogram-derived features such as median, skewness, entropy; texture features such as contrast, homogeneity, Haralick variance)
Biomarker interpretation	• Directly linked to biological process	• Indirect associations with biological process assumed

*https://ibsi.readthedocs.io/en/latest/; *https://www.lifexsoft.org/; *http://www.eletel.p.lodz.pl/programy/mazda/; *https://nmmitools.org/2019/01/01/pyradiomics/

### Image acquisition and normalisation

An element of diversity of acquisition protocols or machines is advantageous at the discovery phase of data-driven biomarkers so that the identified radiomic signatures used in clinical trials are robust enough across a range of platforms [46]. Datasets utilised for radiomic signature development must be representative of the disease and capture the variability and severity for which they will be used. Within a clinical trials framework, as with previously published recommendations and guidelines [6, 47–49], an optimised tightly controlled standardised imaging protocol ensures image quality (low level of noise, artifact-free, spatial resolution) and stability over time, with known intra- and inter-site reproducibility that does not exceed the expected level of change associated with the trial intervention [50]. Phantom studies are limited for quality control of high-dimensionality information [51] because a suitable phantom would need to exhibit high-dimensionality in a realistic setting and cover the requirements of each type of feature.

Basic methods of image normalisation include pixel size resampling by filtering [52] and/or resampling (rescaling) values with respect to global or local mean and standard deviation of reference image/tissue, or by adjusting the histograms [53]. Normalisation methods affect reproducibility of image features [54, 55]. For second-order statistics features, reduction of matrix dimension post-normalisation is needed. This is achieved by discretisation (quantisation, grey-level resampling, histogram re-binning) and reduces noise from clustered intensity values. Choice of the absolute (fixed bin size) or the relative (fixed bin number) method significantly affects the values of texture features and requires optimisation depending on the clinical task at hand [56–58]. Shape features (area, centroid, perimeter, roundness, Feret’s diameter) are less sensitive to differences in intensity values. Both types of features remain dependent on the spatial resolution of the image. Numerical harmonisation of features as an alternative to standardisation of image acquisition and pre-processing is based on transformation of variable feature distributions to a common batch-effect free reference space, to deal with varying imaging conditions [59, 60]

The Image Biomarker Standardization Initiative (IBSI) [61] offers a common reference of definitions and benchmarking of radiomic features and provides recommendations for comprehensive reporting of image acquisition parameters and pre-processing methods.

### Image analysis—segmentation

As with biologically driven biomarkers, manual region of interest delineation introduces inter- and intra-observer variability because of variation in border perception. Observer training and working to protocol assists in this regard. Semi-automated segmentation methods, e.g. region-growing or level set active contour models [62] and deep learning methods [63], are more reproducible [64], but they are dependent on their training set, which may introduce other errors. Quantitative verification metrics [65], such as Dice coefficient, and Hausdorff distance metrics, help determine segmentation reproducibility. Images that require alignment for different time series data, parametric maps and modalities should evaluate deviations in locations (distance) of pairs of homologous landmark points, especially important for non-rigid image registration [66, 67].

### Image analysis—feature extraction

‘Hand-crafted’ radiomics extracts predefined human-engineered features from the volume-of-interest (VOI) [17]. These include shape characteristics, intensity histogram metrics and texture parameters (local binary patterns, grey-level co-occurrence, run-length, zone-length and neighbourhood different matrices, auto-regressive model, Markov random fields, Riesz wavelets, S-transform, fractals) which require specific assumptions in their computation, so that software implementations on different platforms (even if all are IBSI compliant) and between different versions of the same software can lead to different results [68]. Recommendations on calculating and reporting radiomic features have been proposed, and both mathematical equations and pre-processing applied should be reported. The information and framework provided through IBSI [61] should also be followed as much as possible to ensure the quality and relevance of the post-processing (denoising, resampling, enhancement, spatial alignment correction, segmentation and feature extraction). Other descriptive (radiologist-scored), functional (SUV, ADC, K^trans^) or clinical parameters may be added to the radiomic signature if pertinent.

### Computational statistics—feature selection

Several tools are described [69–72]. To identify relevant, non-redundant and stable features with which to build models, three categories of technique are employed. Filter methods (ANOVA, correlation, RELIEF [73]) rely on a criterion function, have low computational cost and are less prone to overfitting, by separating selection from model building; however, they are more unstable to different datasets. Wrapper methods (forward selection, backward elimination, stepwise selection) incorporate a specific machine learning algorithm to eliminate features but have increased computational cost and high probability of overfitting, since model training uses feature combinations that include common features. Embedded methods (LASSO, RIDGE regression) embed features successively and penalise the coefficients of a model that contribute to overfitting at each iteration. They represent a trade-off between filter and wrapper methods.

### Computational statistics—classifier/model

After dimension reduction, selected features are investigated for their association with clinical outcome using tools such as univariable or multivariable logistic regression, decision tree, random forest, support vector machine, neural networks, all described extensively in previous publications [65–68] and used for QIBs and radiomic analyses [24]. Classifiers are differentiated depending on the nature of the clinical outcome, i.e. discrete (mainly binary) or continuous [74, 75]. No tool has proved universally superior and most require a compromise between complexity of tuning versus interpretability of results.

### Computational statistics—deep radiomics (DR)

A recent evolution has been the integration of radiomics with deep learning (DL) [76–78]. ‘Discovery Radiomics’ automatically extracts deep features relevant to a given query (e.g. diagnosis, prognosis) from the data, and the resulting trained model can be applied to complete datasets, avoiding the error-prone segmentation step. As DL can include multiple data types, relevant information in electronic patient records can be exploited.

## Validating the radiomics output

### Technical validation

Following identification of a radiomics signature associated with disease/outcome, two fully independent datasets are needed, one for training and cross-validation (internal validation), and at least one other to test the final model and confirm generalisability and performance (external validation). Both training and testing datasets should be of sufficient uniform quality (data balancing) and representative for the patient population for which the radiomics model is intended. An adequate sample (size and diversity) is essential for the training and validation datasets, with respect to the number and type of features (‘signature’) considered. Testing the model with a dataset containing a different prevalence of cases and/or a high degree of imbalance may result in overoptimistic conclusions. Feature selection avoids over-parameterised models, reduces dimensionality of the feature space (data dimension reduction) and ensures that only a small and stable subset of original features relevant to the task are retained. A strategy to cross-validate the structure of the model requires careful considerations regarding sample size, accuracy estimation and the choice of the validation method (hold-out, k-fold cross-validation, bootstrap). Grid searches pose the danger of overfitting, leading to overoptimistic model performance that is not reproduced on other datasets or in clinical practice. Finally, repeatability and reproducibility of the signature in a multicentre context (affected by imaging apparatus, acquisition protocols and analysis methods) is a crucial step in technical validation [79–81]. As with QIBs, radiomics models should be tested with cross-institutional clinical training and testing datasets to guarantee generalisability to representative patient populations.

### Biological validation

Biological correlation with liquid/tissue biopsies may be performed after the technical and clinical validity of a radiomic signature is established but is not mandatory. A radiomic signature that is related to survival outcomes may potentially reflect a tissue phenotype associated with a specific biology. Biological validation reduces the likelihood that radiomic features are selected by statistical chance or may be attributed to the nature of the data sample used for model development. It also offers the opportunity to reduce the number of selected features.

### Clinical validation

The process by which the clinical utility of a single quantitative feature, or multiple features embedded in a statistical model is demonstrated, allowing improvement of health outcomes (improved diagnosis or therapeutic management of a disease or individual patient) is being addressed slowly for radiomics. Following initial ‘discovery’, new and independent datasets are required to replicate the performance of the identified model and validate it clinically. Performance metrics, e.g. sensitivity and specificity, should be evaluated ideally in prospective trials, or prospectively in the clinic using routinely obtained clinical data (real-life conditions) in order to avoid bias. Table 2 lists some exemplar studies and their clinical use. Broadly speaking, standard recommendations for clinical validation and clinical utility assessment of any QIB should be followed and applied.

**Table 2:** Exemplar radiomics signature studies and their clinical use

Radiomic analysis	Radiomic feature (process)	Modality	Tissue types investigated	Decision-making role
Second-order statistics	Textural (Haralick, Gabor)	CT [29–31] MRI [24–26] PET/CT [37–42]	Lung, breast, brain, liver, prostate, head and neck, lymph node, cervix	• Prognostic • Predictive • Response • Survival • EGFR expression • p53 mutation status
Higher-order statistics Filter grids extract repetitive or non-repetitive patterns	Wavelets	CT [82–87] MRI [88–90] PET/CT [91, 92]	Lung, oesophagus, brain, pancreas, breast, head and neck	• Diagnostic • Prognostic • Predictive • Response • Survival • Surgical resection margins
	Laplacian transforms (bandpass filters)	CT [93, 94] MRI [95–97] PET/CT [92]	Brain, lung, rectum, cervix, kidney	Prognostic Response
	Minkowski functions (patterns of voxels with intensity above threshold)			
	Fractal dimensions (patterns imposed on image and number of grid elements containing voxels of a specified value is computed)			
Delta radiomics	Change in radiomic features	PET/CT [98, 99]	Lung	Response
Dynamic radiomic studies	Pharmacokinetic radiomic features	PET/CT [100]	Lung	Response, data highly correlated to data from static studies

## Biological correlates of radiomic features

Images provide an averaged macroscopic view (with large partial volume effects, both in space and time) of the geometry and/or function of the tissue. Radiomic features are statistical descriptors characterising the macroscopic visual aspect of images and only indirectly relate to the microscopic histological characteristics of the imaged tissue. Such features are then used as a statistical/phenomenological description of the outcome, and not embedded into an actual biological/physical model of this outcome that would unambiguously establish causality between features and outcome.

Radiomic information on visually imperceptible phenotypic characteristics such as intensity, shape, size and texture distinguish benign and malignant tumours, likely reflecting different cellular morphology [101]. In cervix cancer, radiomic features of low-volume tumours with radiomic profiles similar to high-volume tumours had a worse prognosis implying a more aggressive phenotype at an earlier stage [36]. In a lung cancer study, texture entropy and cluster features, as well as voxel intensity variance features, were associated with the immune system, the p53 pathway, pathways involved in cell cycle regulation [102] and for predicting EGFR mutation status [103]. Nevertheless, why specific features are associated with specific pathways remains unexplored and the relationship between radiomic signature and cell morphology, density, distribution pattern, alignment and organelle composition need further elucidation.

Although it is possible to extract mathematically hundreds or thousands of radiomic features from digital images, most studies to date suggest that less than 20 are indicative of unfavourable biology, and these largely relate to shape and textural uniformity. 2D shape features indicate more rapidly progressive disease with reduced overall survival in glioblastoma multiforme [104]. Shape and textural features from CT scans of lung cancer have been shown to predict unfavourable biology (nodal and distant metastases respectively) [105]. In prostate cancer, Gabor textural features (defining spatial frequency patterns within the image) were predictive of Gleason grade on MRI. As gland lumen shape features relate to Gleason grade, discriminability of Gabor features is a likely consequence of variations in gland shape and morphology at the tissue level [106]. In future, prospective selection of a handful of relevant features should become possible to interrogate specific biological processes and pathways being manipulated within clinical trials so that it may be possible for the clinical question to drive the choice of biomarker usage and analysis. However, understanding the biological basis for a biomarker to facilitate its acceptance into clinical practice is not the primary objective of a data-driven process such as radiomics. It may well be that reliable modelling of the outcome with a relatively high and clinically acceptable performance means that biological validation would not be a primary concern [107].

## Limitations of data-driven processes

When defining training datasets for radiomic feature extraction and selection in clinical trials, case-control data may be considered but may underrepresent the disease. Enrichment of training datasets with normal and abnormal cases of varying disease severity is mandatory to achieve appropriate balance. Bias in the training datasets limits generalisability. For example, a radiomic signature developed on lung nodules detected on chest x-rays in a population with a high prevalence of tuberculosis and few cancers will overdiagnose tuberculosis in a population with a high prevalence of cancer. Image acquisition bias (cases recognised as disease acquired with a specific protocol or device) where selected features are linked to image acquisition rather than to image content may fail to predict disease when applied to an independent population. Manual VOI segmentation and use of locally developed methodology risks discovery of features that are not generalisable and may be influenced by hardware or software-related factors rather than the disease itself. Diverse but balanced image acquisition conditions in the training dataset should counteract these effects. Though balance and diversity are necessary at the discovery stage, it is crucial to evaluate performance only on populations representative of the natural prevalence.

The radiomic process, which tests combinations of hundreds and thousands of parameters, risks false discovery. Traditional statistical corrections for multiple tests would lead to *p* values impossible to reach. Strategies to reduce spurious correlations and overfitting include artificially increasing the number of samples by data augmentation (datasets flipped, rotated and deformed to simulate new patients). Cross-validation or bootstrapping are alternative strategies, but an independent dataset to confirm the findings is always required.

## Implementation of radiomics in clinical trials

Although the discovery phase requires image acquisition diversity, standardised protocols, pre- and post-processing methods, tools and algorithms for feature extraction are needed for incorporating into clinical trials and facilitated by centralised data analyses and publicly available analysis software (Table 3). To incorporate radiomics in clinical trials, three potential scenarios can be considered. *Firstly*, where radiomic signature discovery is the objective, a trial should follow the steps described and illustrated (Fig. 2). *Secondly*, a radiomic ‘exploratory end-point’ may form an ancillary study within an established trial. Here, a two-phase process would involve an initial phase utilising more than two-thirds of the final cohort data (training cohort) to identify the most promising feature(s) and a subsequent phase using the remaining patients (independent cohort) to evaluate the performance of the identified radiomic signature. *Thirdly*, where a previously validated radiomic signature is used, this could be incorporated into a clinical trial as a primary or secondary endpoint. In this last case, the pathway of a data-driven biomarker does not differ from a QIB.

**Table 3:** Recommended process for inclusion of data-driven biomarkers into clinical trials

Step	Recommended process for clinical trial inclusion
Image acquisition	Standardised protocol agreed with site with vendor-specific amendments (incl. software version control) to achieve reproducibility of other QIBs within accepted published standards
Image acquisition—normalisation	Raw data saved. Image normalisation predefined
Image analysis—segmentation	If manual or semi-automated, done by centralised/core laboratory by > 1 observer to establish reproducibility. If automated, can be done with CE-marked software with established limits of agreement at local sites
Image analysis—feature extraction	Use of validated features with established error margins, adapted for individual situations. Discard redundant features. Test reproducibility, repeatability within trial setting
Computational statistics—feature and model selection	Based on performance by association with trial endpoint (e.g. response/survival)
Validation	Adequate sample size, test data on samples with similar characteristics, cross-validation strategies, avoid over-fitted models
Biomarker interpretation	Association with positive diagnosis, prognosis or outcome

## Summary and future perspective

Data-driven imaging biomarkers provide information beyond that perceived by human readers. Their benefits may be exploited if specific standardisation and validation pathways are defined and the different/additional hurdles compared to more traditional QIBs are addressed. Effects of different types of processing on subsequent extracted feature variability and predictive model performance is an open area of research [13]. Availability of public access patient cohorts with well-documented image datasets is expected to facilitate consensus regarding pre- and post-processing methods and determine utility of radiomics within clinical trials.

While radiomics may eventually encompass all quantitative image-derived information into a common framework, current implementations mostly relate to intensity, shape and textural features within a VOI. In the future, quantitative (or even qualitative) functional information, e.g. derived from PET, SPECT, pharmacokinetic modelling and other parametric imaging modalities, may form part of the radiomic signature, and require a smaller or biologically more meaningful set of parameters. Deep radiomics may also be deployed in trials, and recent studies have already demonstrated the potential of such approaches [108–111].

Regardless of definitive biological correlation, once adopted and properly deployed, data-driven biomarkers may be combined with clinical data and other biomarkers (biochemical, genetic, epigenetic, transcription factors, proteins). Such expanded use of radiomics should eventually improve disease characterisation, prognostic stratification and response prediction in clinical trials, ultimately advancing precision medicine.

## Abbreviations

ADC: Apparent diffusion coefficient
CE: Conformite Europeenne
CNN: Convolutional neural networks
CT: Computerised tomography
DL: Deep learning
DR: Deep radiomics
EGFR: Epidermal growth factor receptor
FDG: Fluorodeoxyglucose
IBSI: Image biomarker standardisation initiative
MeSH: Medical Subject Headings
MRI: Magnetic resonance imaging
PET: Positron emission tomography
QA: Quality assurance
QC: Quality control
QIBs: Quantitative imaging biomarkers
SPECT: Single photon emission computed tomography
SUV: Standardised uptake value
VOI: Volume of interest
