# Correction to: Incorporating radiomics into clinical trials: expert consensus endorsed by the European Society of Radiology on considerations for data-driven compared to biologically driven quantitative biomarkers

**DOI:** 10.1007/s00330-021-07721-3

**Published:** 2021-03-10

**Authors:** Laure Fournier, Lena Costaridou, Luc Bidaut, Nicolas Michoux, Frederic E. Lecouvet, Lioe-Fee de Geus-Oei, Ronald Boellaard, Daniela E. Oprea-Lager, Nancy A Obuchowski, Anna Caroli, Wolfgang G. Kunz, Edwin H. Oei, James P. B. O’Connor, Marius E. Mayerhoefer, Manuela Franca, Angel Alberich-Bayarri, Christophe M. Deroose, Christian Loewe, Rashindra Manniesing, Caroline Caramella, Egesta Lopci, Nathalie Lassau, Anders Persson, Rik Achten, Karen Rosendahl, Olivier Clement, Elmar Kotter, Xavier Golay, Marion Smits, Marc Dewey, Daniel C. Sullivan, Aad van der Lugt, Nandita M. deSouza, European Society of Radiology

**Affiliations:** 1 PARCC, INSERM, Radiology Department, AP-HP, Hopital europeen Georges Pompidou, Université de Paris, F-75015 Paris, France; 2 European Imaging Biomarkers Alliance (EIBALL), European Society of Radiology, Vienna, Austria; 3 Imaging Group, European Organisation of Research and Treatment in Cancer (EORTC), Brussels, Belgium; 4 School of Medicine, University of Patras, University Campus, Rio, 26 500 Patras, Greece; 5 College of Science, University of Lincoln, Lincoln, LN6 7TS UK; 6 Department of Radiology, Institut de Recherche Expérimentale et Clinique (IREC), Cliniques Universitaires Saint Luc, Université Catholique de Louvain (UCLouvain), B-1200 Brussels, Belgium; 7 Department of Radiology, Leiden University Medical Center, Leiden, The Netherlands; 8 Biomedical Photonic Imaging Group, University of Twente, Enschede, The Netherlands; 9 Department of Radiology & Nuclear Medicine, Cancer Centre Amsterdam, Amsterdam University Medical Centers (VU University), Amsterdam, The Netherlands; 10 Quantitative Imaging Biomarkers Alliance, Radiological Society of North America, Oak Brook, IL USA; 11 Department of Quantitative Health Sciences, Cleveland Clinic, Cleveland, OH USA; 12 Department of Biomedical Engineering, Istituto di Ricerche Farmacologiche Mario Negri IRCCS, Bergamo, Italy; 13 Department of Radiology, University Hospital, LMU Munich, Munich, Germany; 14 Department of Radiology & Nuclear Medicine, Erasmus MC, University Medical Center, Rotterdam, The Netherlands; 15 Division of Cancer Sciences, University of Manchester, Manchester, UK; 16 Department of Biomedical Imaging and Image-guided Therapy, Medical University of Vienna, Vienna, Austria; 17 Department of Radiology, CentroHospitalar Universitário do Porto, Instituto de Ciências Biomédicas de Abel Salazar, University of Porto, Porto, Portugal; 18 Quantitative Imaging Biomarkers in Medicine (QUIBIM), Valencia, Spain; 19 Nuclear Medicine, University Hospitals Leuven, Leuven, Belgium; 20 Nuclear Medicine and Molecular Imaging, Department of Imaging and Pathology, KU Leuven, Leuven, Belgium; 21 Division of Cardiovascular and Interventional Radiology, Dept. for Bioimaging and Image-Guided Therapy, Medical University of Vienna, Vienna, Austria; 22 Department of Radiology and Nuclear Medicine, Radboud University Medical Center, 6525 GA Nijmegen, The Netherlands; 23 Radiology Department, Hôpital Marie Lannelongue, Institut d’Oncologie Thoracique, Université Paris-Saclay, Le Plessis-Robinson, France; 24 Nuclear Medicine, Humanitas Clinical and Research Hospital – IRCCS, Rozzano, MI Italy; 25 Imaging Department, Gustave Roussy Cancer Campus Grand, Paris, UMR 1281, INSERM, CNRS, CEA, Universite Paris-Saclay, Saint-Aubin, France; 26 Department of Radiology, and Department of Health,Medicine and Caring Sciences, Center for Medical Image Science and Visualization (CMIV), Linköping University, Linköping, Sweden; 27 Department of Radiology and Medical Imaging, Ghent University Hospital, Gent, Belgium; 28 Department of Radiology, University Hospital of North Norway, Tromsø, Norway; 29 Department of Radiology, University Medical Center Freiburg, Freiburg, Germany; 30 Queen Square Institute of Neurology, University College London, London, UK; 31 Department of Radiology, Charité Universitätsmedizin Berlin, Berlin, Germany; 32 Dept. of Radiology, Duke University, 311 Research Dr, Durham, NC 27710 USA; 33 Division of Radiotherapy and Imaging, The Institute of Cancer Research and Royal Marsden NHS Foundation Trust, London, UK; 34 Am Gestade 1, 1010 Vienna, Austria

**Correction to: European Radiology**

10.1007/s00330-020-07598-8

The original version of this article, published on 25 January 2021, unfortunately contained mistakes. The following corrections have therefore been made in the original: Firstly, “endorsed by the European Society of Radiology” was missing in the article title. Secondly, the institutional author “European Society of Radiology” was missing in the author line, including the related affiliation 34. Thirdly, the following sentence was missing in the Acknowledgements: This paper was endorsed by the ESR Executive Council in December 2020. The corrected title and author line are given above; the corrected affiliations are given below. The original article has been corrected.

